# Rusticus in Town (Poetical)

**Published:** 1874-08

**Authors:** 


### Our Exchanges.

### RUSTICUS IN TOWN.

The following poem was read at the annual meeting of the Massachusetts Medical Society, June 3, 1874, by Dr. Thomas N. Stone, of Wellfleet:

"Again we rest, as well we need,"
I said last week to my old steed.
I always talk in a brotherly way
To that same steed, so staid and gray;
For, though small faith in Darwin's creed,

So much good lies in that steed,
It may outlive the stroke of death,
And show some soul beyond mere breath.

My good gray heard, with ears attent,
As if she knew what each word meant;
She smiled in a sad and anxious way,
As to the cars I turned away,
As if she would in warning say,
"Good master of thyself take heed—
No love like mine has iron steed."

I tread your streets in country style,
O'er paths of brick, 'neath granite pile,
To stores where health is sold;
To learn if, perchance, some chemistry rare
Had added strength to God's own air
Our father's breathed of old.

As I go prowling round, no doubt
Clerks think that Rusticus is out,
Or some poor country cuss,
Whose head no beaver ever pressed,
Whose hand no kid glove e'er caressed,
Who never rode in a buss.

As 'fore some label long I stop,
I scratch my pate till hay seeds drop
From out my tangled hair.
Here lacto-peptine, thund'ring word,
Does every power of Nature hoard;
No mortals need despair.

Pancreatine our lacteals play
So gently on our festal day,
No colic need we fear.
No longer now for costive ills,
Need we resort to nauseous pills—
The *prima via's* clear.

This nervous force of Brown-Sequard
We need no longer now regard;
'Tis furnished by strychnine.
If lungs and liver both are gone,
We'll make the patient still live on
By cod oil *et* pepsin.

The fruit is from the tree of life
Old Adam lost through foolish strife,
And Eve forgot to steal.
Where's Holmes, who says there is no cure
But poppy-leaves and quinine pure
Our mortal ills to heal?

If death should travel now our vale
To seize consumption's victims pale,
 We've got a dose divine.
I tried it on my skeleton,
And soon upon each naked bone
 Muscles began to shine.

Its cod oil greased each stiffened joint,
And rounded well each angle's point,
 While phosphorous made his brain.
Beef gave the food he'd wanted long;
Its wine and iron made him strong—
 The rascal broke his chain!

He took my cloak from off the wall,
My beaver from the outer hall,
 And then he took his way.
While I, confounded at his brass,
Just stepped aside to let him pass,
 Nor dared I say him nay,

A wand'ring Jew he walks the earth,
For where's the spot that gave him birth,
 His new brain can't divine.
If such an one, in daily round,
By any country brother's found,
 Please take that hat of mine.

